# Impact of perinatal antibiotic exposure on the infant gut microbiota at one year of age

**DOI:** 10.1186/1710-1492-10-S1-A31

**Published:** 2014-03-03

**Authors:** Ryan Persaud, Meghan B Azad, Theodore Konya, David S Guttman, Radha S Chari, Malcolm R Sears, Allan B Becker, James A Scott, Anita L Kozyrskyj

**Affiliations:** 1 Pharmacy, University of Manitoba, Winnipeg, Manitoba, Canada; 2 Pediatrics, University of Alberta, Edmonton, Alberta, Canada; 3 Dalla Lana School of Public Health, University of Toronto, Toronto, Ontario, Canada; 4 Cell & Systems Biology, University of Toronto, Toronto, Ontario, Canada; 5 Obstetrics & Gynecology, University of Alberta, Edmonton, Alberta, Canada; 6 Department of Medicine, McMaster University, Hamilton, Ontario, Canada; 7 Pediatrics & Child Health, University of Manitoba, Winnipeg, Manitoba, Canada; 8 Canadian Healthy Infant Longitudinal Development Study

## Background

Disruption of the infant gut microbiota has been linked to long-term health outcomes, including obesity and allergic disease [1]. Antibiotics can significantly alter the microbiota [2], but evidence is scarce for the long-term effect of antibiotic exposure during the perinatal period (shortly before or after delivery). Our research aimed to determine the impact of infant and maternal perinatal antibiotic exposure on the infant gut microbiota at 1 year of age.

## Methods

Antibiotic exposure in the perinatal period was documented by retrospective hospital chart review of 184 Manitoban infants enrolled in the Canadian Healthy Infant Longitudinal Development (CHILD) Study. Data on mode of delivery, indications for treatment, and maternal health status were also collected. Gut microbiota composition (relative abundance of select taxa chosen for their previous association with antibiotic exposure or health outcomes) was determined by Illumina 16S rRNA sequencing of fecal samples collected at 1 year of age. Microbiota diversity was characterized using Chao1 Richness Estimator, Shannon Diversity Index, and Simpson Diversity Index.

## Results

Of the 184 infants, 8 (4.4%) received intravenous antibiotics during the perinatal period for suspected sepsis. Seventy-two (39.1%) mothers received antibiotics shortly before or after delivery, resulting in indirect antibiotic exposure of the infant via placental transfer or breast milk. Indirect perinatal antibiotic exposure did not alter the 1 year old infant’s overall microbiota diversity; however, these infants had an increased relative abundance of *Clostridium* (p=0.04). Infants who were both directly and indirectly exposed to antibiotics had increased microbiota diversity at 1 year of age (p<0.03 for Shannon and Simpson diversity indices computed at Family and Genus levels). These infants also had a lower relative abundance of the phylum Bacteriodetes (p=0.03), and increased relative abundance of the genus *Akkermansia* (p=0.03) and the phylum Proteobacteria (p=0.02). Similar trends were observed following stratification by mode of delivery, premature rupture of membranes, presence of Group B *Streptococcus*, breastfeeding, and postnatal antibiotics (throughout the first year); except for the decrease in Bacteroidetes, which was attenuated in breastfed infants.

## Conclusions

Antibiotics administered at birth are associated with long-lasting changes to the infant gut microbiota, increasing overall diversity and modifying community composition until at least 1 year of age. Maternal perinatal antibiotics may also influence infant gut microbiota composition. Going forward, we will conduct comprehensive microbiota analyses and investigate how antibiotic-associated changes ultimately influence health outcomes during later childhood.

## References

[B1] Penders J Thijs C van den Brandt PA Kummeling I Snijders B Stelma F Adams H van Ree R Stobberingh EE Gut microbiota composition and development of atopic manifestations in infancy: the KOALA Birth Cohort Study Gut 2007 10 661 7 17047098 10.1136/gut.2006.100164 PMC1942165

[B2] Willing BP Russell SL Finlay BB Shifting the balance: antibiotic effects on host–microbiota mutualism Nature Reviews Microbiology 2011 10 233 243 21358670 10.1038/nrmicro2536

